# Targeting of Epithelial Cell Adhesion Molecule-Expressing Malignant Tumors Using an Albumin-Binding Domain-Fused Designed Ankyrin Repeat Protein: Effect of the Molecular Architecture

**DOI:** 10.3390/ijms26115236

**Published:** 2025-05-29

**Authors:** Vladimir Tolmachev, Anzhelika Vorobyeva, Alia Hani Binti Rosly, Javad Garousi, Yongsheng Liu, Torbjörn Gräslund, Eleftherios Papalanis, Alexey Schulga, Elena Konovalova, Anna Orlova, Sergey M. Deyev, Maryam Oroujeni

**Affiliations:** 1 Department of Immunology, Genetics and Pathology, Uppsala University, 751 85 Uppsala, Sweden; anzhelika.vorobyeva@igp.uu.se (A.V.); alia-hani-binti.rosly.1042@student.uu.se (A.H.B.R.); yongsheng.liu@igp.uu.se (Y.L.); eleftherios.papalanis@igp.uu.se (E.P.); maryam.oroujeni@igp.uu.se (M.O.); 2 Department of Protein Science, KTH—Royal Institute of Technology, 106 91 Stockholm, Sweden; garousi@kth.se (J.G.); torbjorn@kth.se (T.G.); 3 Molecular Immunology Laboratory, Shemyakin and Ovchinnikov Institute of Bioorganic Chemistry, Russian Academy of Sciences, 117997 Moscow, Russia; schulga@gmail.com (A.S.); elena.ko.mail@gmail.com (E.K.); biomem@mail.ru (S.M.D.); 4 Research Centrum for Oncotheranostics, Research School of Chemistry and Applied Biomedical Sciences, Tomsk Polytechnic University, 634050 Tomsk, Russia; 5 Department of Medicinal Chemistry, Uppsala University, 751 83 Uppsala, Sweden; anna.orlova@ilk.uu.se

**Keywords:** designed ankyrin repeat protein, epithelial cell adhesion molecule (EpCAM), albumin-binding domain (ABD), fusion protein, nude mice, xenograft

## Abstract

Designed ankyrin repeat protein (DARPin) Ec1, a small scaffold protein (18 kDa), binds with high affinity the epithelial cell adhesion molecule (EpCAM) that is overexpressed in several carcinomas. To enhance the targeted delivery of cytotoxic drugs using Ec1, we investigated the potential of fusing Ec1 with an albumin-binding domain (ABD) to improve its circulation time and decrease renal uptake. Two fusion proteins were created, Ec1-ABD, with the ABD at the C-terminus, and ABD-Ec1, with the ABD at the N-terminus. Both variants were labeled with ^111^In. ABD-fused variants bound specifically to EpCAM-expressing cells with picomolar affinity. Adding human albumin reduced the affinity. This effect was more pronounced for Ec1-ABD; however, the affinity remained in the subnanomolar range. The position of the ABD did not influence the internalization rate of both variants by human cancer cells. In mouse models with human cancer xenografts, both variants demonstrated over 10-fold lower renal uptake compared to the Ec1. Tumor uptake of the ABD-fused variants was higher than the uptake of Ec1. ABD-Ec1 provided two-fold higher tumor uptake, indicating fusion with an ABD as a promising way to modulate the targeting properties of an Ec1-based construct. However, the effect of fusion depends on the order of the domains.

## 1. Introduction

The progress in treating metastatic malignant tumors during recent years has been associated with an increasing application of antibody–drug conjugates (ADCs) [1,2]. While these targeted anticancer drugs provide clear benefits of increased survival and reduced off-tumor toxicities, the production and use of antibodies are associated with some challenges, such as, e.g., costly production in mammalian cells due to a complex pattern of disulfide bonds and glycosylation [3]. The combination of light and heavy chains in immunoglobulins necessitates the use of complex genetic expression constructs and complicates the development of fusion proteins with pre-determined structures and pharmacologic properties [3]. A possible solution to bypass these obstacles is to use engineered scaffold proteins, which can be selected for a specific high-affinity binding to cancer-associated proteins [3,4,5,6]. Designed ankyrin repeat proteins (DARPins) are a class of engineered scaffold proteins. The DARPin scaffold is composed of four or five repeats containing 33 amino acids each. A part of the amino acids can be randomized to establish combinatorial libraries enabling the selection of high-affinity binders [7,8]. DARPins binding to such tumor-associated molecular targets, such as human epidermal growth factor receptor 2 (HER2), epithelial cell adhesion molecule (EpCAM), epidermal growth factor receptor (EGFR), vascular endothelial growth factor (VEGF), and hepatocyte growth factor (HGF) have been described [9,10,11,12]. The attractive features of DARPins as tumor-targeting agents include their stability, high water solubility, cysteine-independent folding, and low-cost production in non-mammalian cells [7,8]. Clinical trials have demonstrated that DARPin-based radionuclide imaging probes can successfully target and visualize cancer-associated upregulation of proteins in malignancies [13,14].

A promising and druggable cancer-associated molecular target is the epithelial cell adhesion molecule (EpCAM, also known as CD326). EpCAM is a transmembrane glycoprotein that is involved in signaling regulating migration, proliferation, and differentiation [15]. EpCAM is overexpressed in different malignancies such as prostate, ovarian, lung, colon, and breast cancers [16,17]. Several therapeutic approaches for targeting EpCAM in different cancers using antibody derivatives have been evaluated in preclinical models and in clinical trials [18,19,20,21,22]. The DARPin Ec1 (five ankyrin repeats, molecular weight 18.6 kDa) binds to EpCAM with high affinity (K_D_ = 68 pM) [10]. Radiolabeling of Ec1 with different nuclides has enabled the development of radionuclide imaging probes demonstrating specific visualization of EpCAM expression in preclinical models of pancreatic, ovarian, prostate, and triple-negative breast cancers [23,24,25,26]. A Phase I clinical study demonstrated that injections of radiolabeled Ec1 are safe and well tolerated and that the tracer can visualize EpCAM-expressing lung cancers [14]. These data suggest that Ec1 might be used for cancer therapy to deliver cytotoxic payloads to the tumor.

The success of Ec1 as an imaging probe is a consequence of its small size. DARPins pass rapidly through the glomerular membrane in the kidney because their molecular weight is lower than the renal filtration threshold (60 kDa). This minimizes the background caused by a tracer in blood and normal tissue. However, the short biologic half-life in blood causes low tracer bioavailability, resulting in inefficient accumulation in the tumor [4,5]. In addition, the high renal reabsorption of DARPins results in an elevated renal uptake, which would be problematic for therapy using targeted cytotoxic compounds. A potential solution for DARPins and other small targeting agents is the extension of their blood half-life [27]. Extension of the half-life can help avoid infusion or repeated injections of the proteins to maintain high serum concentrations. A possible strategy for the half-life extension is based on a controllable increase of the size to exceed the renal filtration threshold. This might be achieved by conjugation with a large chain of polyethylene glycol (PEGylation) [28] or fusion with unstructured proteins containing hydrophilic amino acids such as Pro, Ala, and Ser (PAS) or Ala, Glu, Gly, Pro, and Ser (XTEN) [29]. An alternative approach is fusion to an engineered scaffold protein, binding to the host’s serum albumin, e.g., an albumin-specific DARPin [30] or an albumin-binding domain (ABD) [31]. The application of these approaches for the half-life extension of DARPins has been quite fruitful. A gene encoding a half-life-extending moiety might be a superior approach compared to conjugation, since it enables the production of the whole targeting constructs in one step, without an additional coupling procedure. Furthermore, our experience with HER2-targeting affibody molecules suggests that the use of an albumin-binding domain (ABD) provides better tumor localization than the use of PAS or XTEN [32]. Most likely, this is the effect of the smaller hydrodynamic radius of the affibody-ABD albumin complex, enabling more efficient extravasation into tumors. However, evaluation of ABD fusions of the HER2-targeting DARPin G3 showed that their affinity to both HER2 and albumin depends on the positioning of ABD relative to DARPin [31]. Thus, the development of an EpCAM targeting system based on Ec1-ABD fusion should include determining the optimal architecture providing the best biodistribution and targeting features.

To evaluate the impact of the domain sequence in an Ec1-ABD fusion on targeting, we used radiolabeling. The use of radioactive labels is a powerful tool in the development of targeted anti-cancer drugs, helping to understand their mechanisms of action and biodistribution at preclinical [29,33,34] and clinical stages [2,35,36]. It permits facile and quantitative evaluation of the concentrations of the targeting agents not only in body fluids but also in solid tissues including tumors. In this study, we used the radionuclide In-111 to evaluate the biodistribution of Ec1-based constructs. This radionuclide has a half-life of 2.8 d, which allows its distribution to be followed for several days. Emission of body-penetrating gamma quanta with energies of 171 (91%) and 245 (94%) keV permits measurement of activity in both ex vivo samples and in vivo. Importantly, In-111 is a residualizing label that remains at the site of the internalization and catabolism of the targeting proteins.

**Figure 1:**
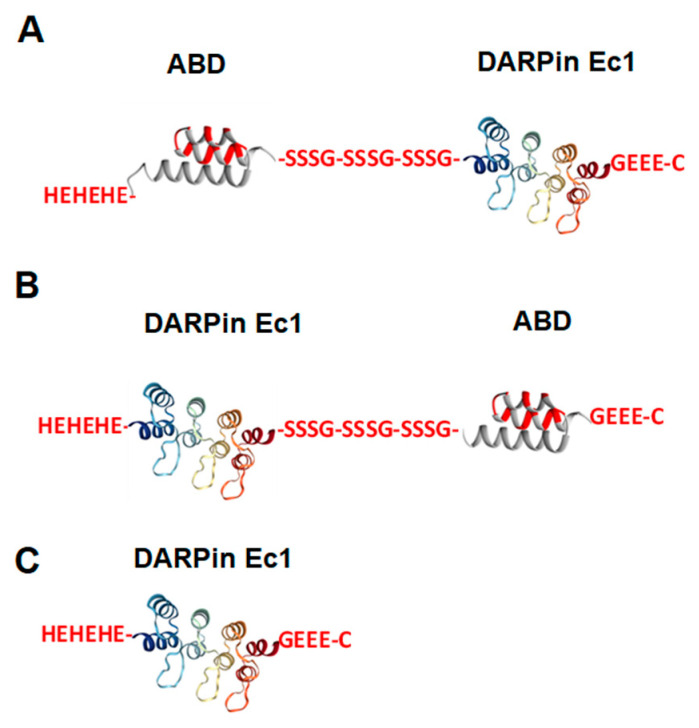
Schematic structures of (**A**) ABD-Ec1, (**B**) Ec1-ABD, and (**C**) non-ABD-fused Ec1 (as a control). All three variants contain a DOTA chelator at the C-terminus for labeling with In-111.

Two variants of fusion proteins (Figure 1) were produced and characterized. In the variant designated ABD-Ec1, the ABD was positioned at the N-terminus (Figure 1A). In the variant Ec1-ABD, ABD was placed at the C-terminus (Figure 1B). The domains were connected by hydrophilic (S_3_G)_3_-linkers, which demonstrated a capacity to prevent mutual steric hindrance in homo- and heterodimeric constructs based on ADAPT6 and ABD [37,38]. Experience showed that the use of shorter interdomain linkers might result in the loss of the helical structure of the domains and less accurate refolding [37]. The N-termini of both constructs contained (HE)_3_-tags, facilitating IMAC purification and improving biodistribution [39]. A single cysteine was introduced at the C-terminus of each construct for site-specific conjugation of a maleimide derivative of the 1,4,7,10-tetraazacyclododecane-1,4,7,10-tetracetic acid (DOTA) chelator to provide a stable radiolabeling with ^111^In. As a non-ABD-fused control, Ec1 containing an N-terminal (HE)_3_-tag and C-terminal DOTA was used. All variants were labeled with In-111. In vitro studies (to test specificity, affinity, and internalization) were performed using human EpCAM-expressing ovarian cancer cells. The blood kinetics of ABD-fused variants were studied in normal NMRI mice. Biodistribution of the ^111^In-labeled DARPin variants was studied in mice bearing EpCAM-expressing SKOV-3 xenografts. Non-ABD-fused DARPin Ec1 was used as a control in this study. Uptake in EpCAM-negative Ramos xenografts was used to test specificity in vivo. SPECT/CT imaging was performed to confirm the biodistribution results.

## 2. Results and Discussion

### 2.1. Production, Purification, and Characterization of ABD-Fused DARPin Ec1 Variants and Ec1 and Conjugation of DOTA Chelator

The constructs were produced and successfully coupled with a DOTA chelator to enable radiolabeling and quantitative evaluation of the biodistribution. According to LC-MS analysis (Supplemental Figure S2), the use of an eleven-fold molar excess of DOTA maleimide for coupling to freshly reduced DARPin provided conjugation of the chelator to all proteins. No unconjugated DARPins were found.

### 2.2. Radiolabeling of Ec1 Variants with ^111^In and In Vitro Stability

The Ec1-derived constructs were then radiolabeled, and the results are presented in Table 1. The radiochemical yield was 91 ± 7 and 61 ± 24% for Ec1-ABD and ABD-Ec1, respectively. After a pre-separation challenge with an excess amount of EDTA, the radiochemical yield dropped to 79 ± 18 and 52 ± 23%, respectively. The size-exclusion purification of free ^111^In was very efficient, and the radiochemical purity of the radiolabeled conjugates after purification using NAP-5 columns was >98%. Radiolabeling of non-ABD-fused Ec1 was performed by the same method as the labeling of ABD-fused Ec1 variants, resulting in a radiochemical purity over 95%.

After the purification, an in vitro stability test of both ABD-fused Ec1 variants (labelled with ^111^In) was performed in PBS and in PBS with a 1000-fold molar excess of EDTA. The release of the ^111^In was slightly higher for Ec1-ABD both in PBS and in PBS with EDTA than for ABD-Ec1. The release indicates that a small fraction of radionuclide was coupled to protein not via DOTA but via a weak chelating site formed by side chains of the amino acids. According to the radio-HPLC radiochromatogram (Supplemental Figure S3), the retention time of the radiolabeled conjugates was around 12 min. Only one major peak was observed, corresponding to the radiolabeled compound. Overall, the labeling of DARPins with ^111^In was reasonably stable, and a simple size-exclusion purification provided high radiochemical purity of the constructs.

### 2.3. In Vitro Studies

**Figure 2:**
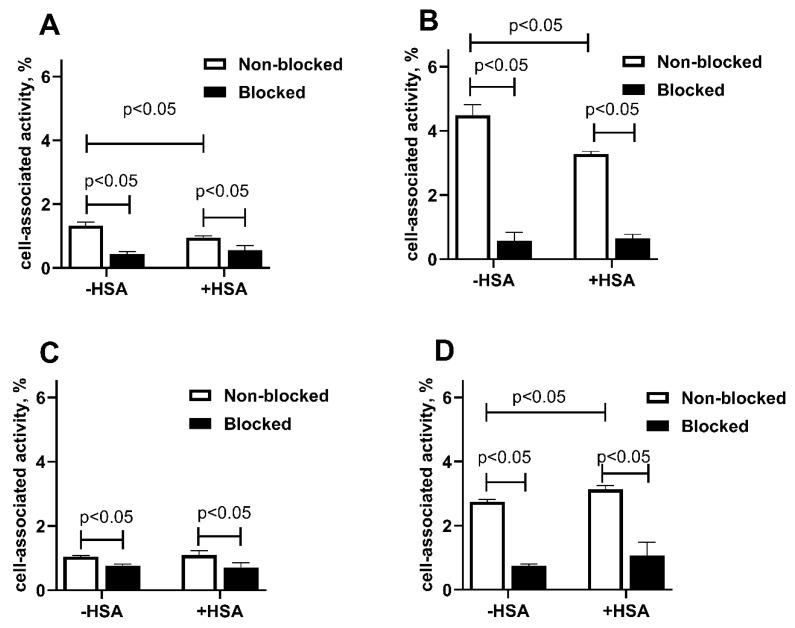
In vitro binding specificity of [^111^In]In-Ec1-ABD (**A**,**B**) and [^111^In]In-ABD-Ec1 (**C**,**D**) on SKOV-3 (**A**,**C**) and OVCAR3 (**B**,**D**) cells. For the blocking of EpCAM receptors, a 100-fold molar excess of a non-labeled Ec1 was added before adding the radiolabeled conjugate. The data are presented as an average value from three samples ± SD.

The specificity of constructs’ binding to living EpCAM-expressing SKOV-3 and OVCAR-3 ovarian cancer cells was investigated by a saturation test. A significant (*p* < 0.05) reduction in cell-associated activity was observed in the case of pre-saturation of EpCAM (blocked groups) for both cell lines, which confirmed the specific binding of both radioconjugates. For [^111^In]In-Ec1-ABD, a significant (*p* < 0.05) reduction in cell-associated activity in the presence and absence of HSA was observed for both cell lines (Figure 2A,B). Thus, in vitro studies demonstrated that both radioconjugates had a preserved and specific binding to EpCAM-expressing cells both in the presence and in the absence of human serum albumin. Still, adding albumin had different effects on the binding level for different constructs. The reduction of [^111^In]In-Ec1-ABD binding in the presence of albumin might indicate a steric hindrance for such binding from the bulky albumin attached to the C-terminus of the targeting molecule. Apparently, this was not the case when the albumin-binding domain was placed at the N-terminus of Ec1.

Moreover, the kinetics of [^111^In]In-ABD-Ec1 and [^111^In]In-Ec1-ABD binding to living SKOV-3 cells was measured using a LigandTracer instrument. The affinity was evaluated by InteractionMap analysis of the LigandTracer sensorgrams. The experiment was performed with or without the addition of HSA (100 nM) to mimic an in vivo milieu and to understand if interaction with HSA influences the binding of the conjugates to EpCAM proteins. The results of the InteractionMap analysis are presented in Table 2. Determination of the kinetic constants showed that the apparent equilibrium dissociation constants (K_D_) values were in the subnanomolar range for binding without adding albumin. The affinity was reduced five-fold in the presence of HSA for [^111^In]In-Ec1-ABD. For [^111^In]In-ABD-Ec1, the reduction was two-fold, and the K_D_ value remained in the subnanomolar range. For both constructs, an addition of albumin increased the dissociation rate.

**Figure 3:**
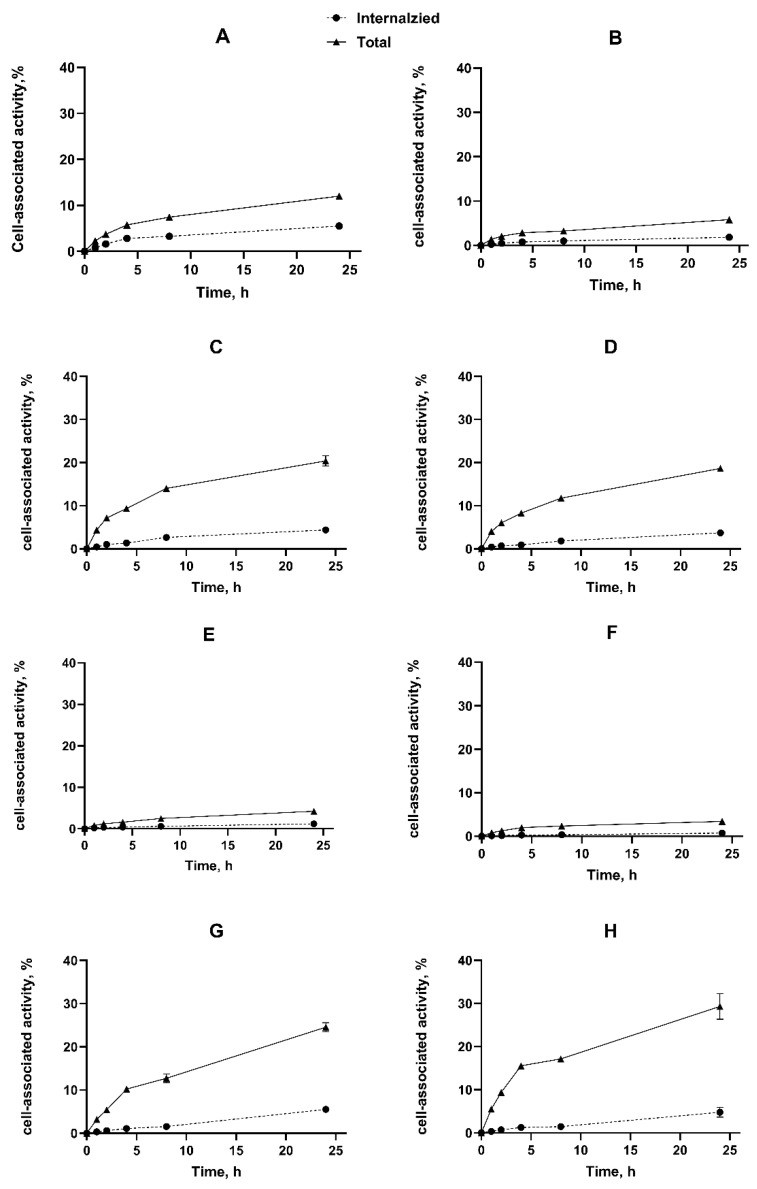
Cellular processing of [^111^In]In-Ec1-ABD (**A**–**D**) and [^111^In]In-ABD-Ec1 (**E**–**H**) by SKOV-3 cells (**A**,**B**,**E**,**F**) and OVCAR3 cells (**C**,**D**,**G**,**H**) without addition (**A**,**C**,**E**,**G**) and with addition (**B**,**D**,**F**,**H**) of HSA (100 nM). Cells were incubated with 2 nM of radioconjugate. The data are presented as an average (*n* = 3) and SD.

The cellular processing data (Figure 3) showed that the pattern of binding and internalization was similar for [^111^In]In-Ec1-ABD and [^111^In]In-ABD-Ec1 for both tested cancer cell lines. The cell-associated activity increased during the incubation. Both constructs were internalized slowly, and approximately 10% of the total cell-associated activity was internalized after 24 h incubation. No significant influence of albumin on internalization was observed. Such an internalization pattern is characteristic for radiolabeled Ec1 derivative internalization by ovarian cancer cells [25]. Interestingly, a fusion of Ec1 with a truncated variant of Pseudomonas exotoxin A (LoPE) was internalized approximately twice as fast [40]. A more rapid in vitro internalization of the Ec1-LoPE fusion was also found in the case of prostate cancer cell lines [41].

Overall, the in vitro studies showed that both [^111^In]In-Ec1-ABD and [^111^In]In-ABD-Ec1 constructs bind specifically to EpCAM-expressing ovarian cancer cells. Adding albumin reduced the binding affinity in a manner that was dependent on the sequence of domains within the constructs. Placement of the ABD at the C-terminus of Ec1, which was the more favorable approach for the HER2-binding DARPin G3 [31], resulted in a stronger reduction of the binding strength to living EpCAM-expressing cells, i.e., was less suitable for this target. The internalization of ABD-fused Ec1 derivatives was slow and independent of the construct’s architecture.

### 2.4. In Vivo Studies

**Figure 4:**
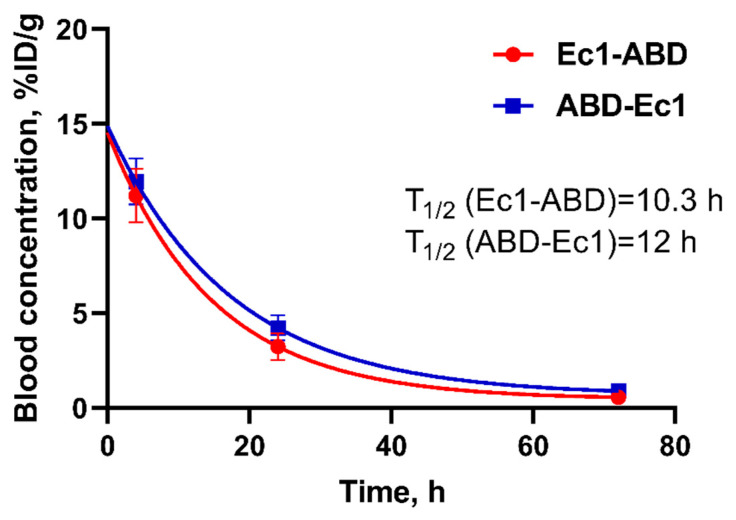
Blood kinetics of ABD-fused Ec1 variants labeled with ^111^In. An amount of 10 µg/30 kBq in 100 µL of 1% BSA in PBS was injected per mouse. Uptake is expressed as %ID/g and presented as the average value from 4 mice ± SD.

The next step was to study the effect of domains sequence on in vivo behavior. The kinetics of [^111^In]In-Ec1-ABD and [^111^In]In-ABD-Ec1 in blood were studied in NMRI mice. The results of this experiment demonstrated that the biological half-life of Ec1-ABD in the blood (T_1/2_ = 10.3 h) was somewhat shorter than the blood half-life of ABD-Ec1 (T_1/2_ = 12 h) (Figure 4).

**Figure 5:**
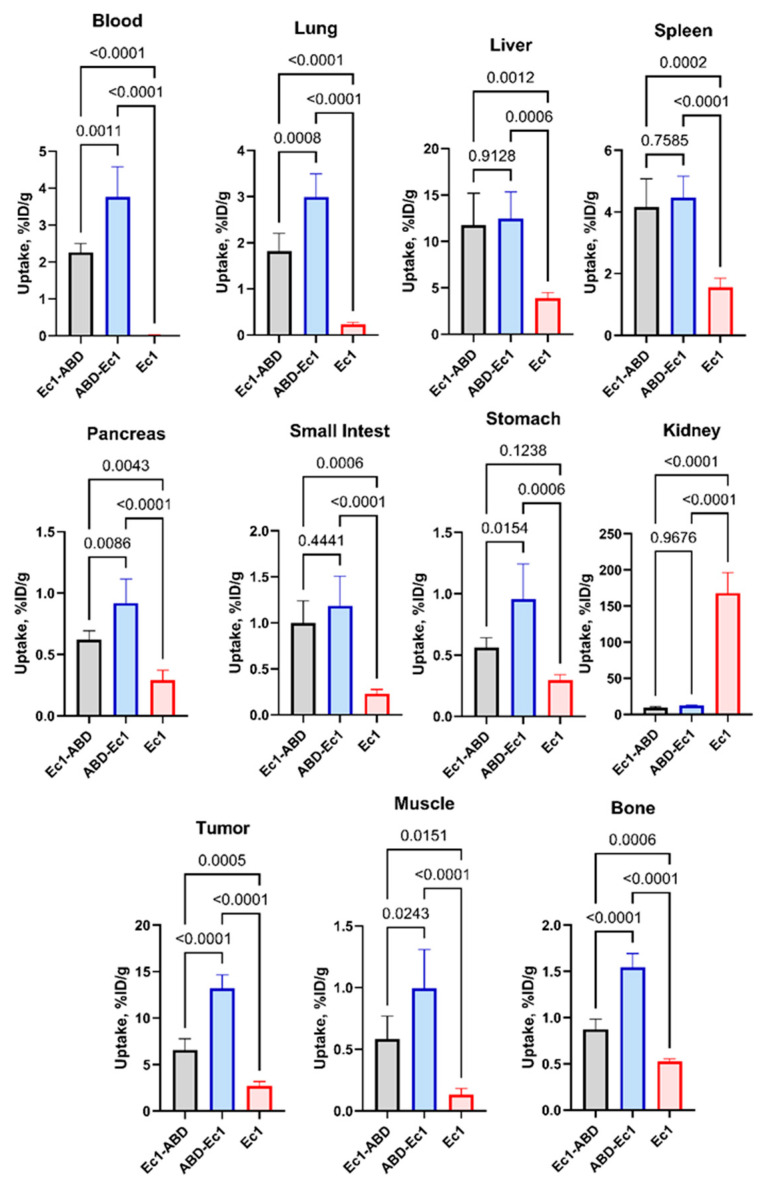
Effect of ABD fusion and construct design on biodistribution of ^111^In-labeled Ec1 derivatives in nude mice bearing SKOV-3 xenografts 48 h after injection. *p*-values are calculated by one-way ANOVA with Tukey correction for multiple comparisons.

The effect of the fusion of DARPin Ec1 with ABD and the impact of the sequence of domains within the constructs on targeting properties was evaluated by measurement of the biodistribution of [^111^In]In-Ec1-ABD, [^111^In]In-ABD-Ec1, and non-ABD-fused [^111^In]In-Ec1 in mice bearing EpCAM-expressing SKOV-3 xenografts 48 h after injection (Figure 5 and Supplementary Table S1). The biodistribution data demonstrated that the fusion with ABD resulted in much higher blood concentration of both ABD-fused variants, [^111^In]In-Ec1-ABD (2.27 ± 0.23%ID/g) and [^111^In]In-ABD-Ec1 (3.76 ± 0.81%ID/g), compared with non-ABD-fused [^111^In]In-Ec1 (0.03 ± 0.01%ID/g). The uptake in most organs and tissues was significantly (*p* < 0.05) higher for the ABD-fused Ec1 variants than for non-ABD-fused Ec1, most likely due to the higher bioavailability of these constructs in the blood. Importantly, the renal uptake was much lower for [^111^In]In-Ec1-ABD (9.6 ± 1.2%ID/g) and [^111^In]In-ABD-Ec1 (12.1 ± 1.1%ID/g) compared with [^111^In]In-Ec1 (168 ± 28%ID/g). The tumor uptakes of both ABD-fused variants were much higher than the uptake of [^111^In]In-Ec1 (2.7 ± 0.5%ID/g). Thus, the fusion of Ec1 resulted in a more than 10-fold reduction of renal uptake and a substantial increase in the accumulation in tumors.

The position of the ABD within the constructs had a clear impact on their biodistribution. [^111^In]In-ABD-Ec1 showed significantly (*p* < 0.05) higher blood concentration than [^111^In]In-Ec1-ABD. The uptake of [^111^In]In-Ec1-ABD was significantly (*p* < 0.05) higher than the uptake of [^111^In]In-ABD-Ec1 in the lung, pancreas, and stomach. The tumor uptake of [^111^In]In-ABD-Ec1 (13.2 ± 1.5%ID/g) was double the uptake of [^111^In]In-Ec1-ABD (6.6 ± 1.2%ID/g).

**Figure 6:**
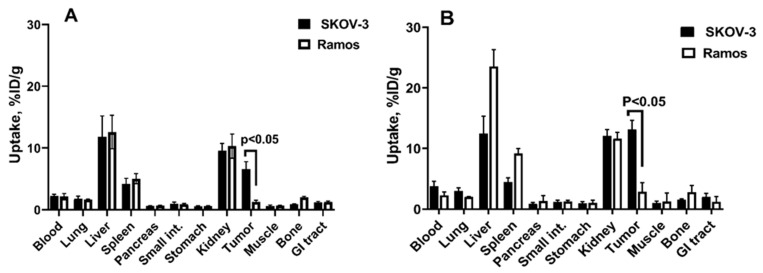
Uptake of (**A**) [^111^In]In-Ec1-ABD and (**B**) [^111^In]In-ABD-Ec1 in SKOV-3 (EpCAM-positive) and Ramos (EpCAM-negative) xenografts at 48 h after injection. Data are expressed as %ID/g and represent averages from four mice ± SD. *p*-value was obtained in an unpaired *t*-test.

To test if the tumor uptake was dependent on the EpCAM expression level, the uptake of [^111^In]In-Ec1-ABD and [^111^In]In-ABD-Ec1 was compared in EpCAM-positive SKOV-3 and EpCAM-negative Ramos tumors (Figure 6). It has to be noted that we used a lymphoma xenograft as a negative control to a carcinoma xenograft. Ideally, one should use an EpCAM-negative tumor of epithelial origin for this purpose. However, EpCAM is expressed in most epithelial organs and in the majority of malignancies of epithelial origin. The EpCAM expression is so common that it is used to identify circulating carcinoma cells in blood samples (see e.g., [42]). Thus, the usage of an EpCAM-negative epithelial tumor xenograft would have required an unusual cell line with cancer-associated mutation irreversibly eliminating the expression of EpCAM. Such a cell line is not available for us. Suppression of EpCAM expression might be also expected in the case of the epithelial-mesenchymal transition. However, this phenomenon is not always retained in tumor xenografts. On the other hand, Ramos xenografts develop tumor microvasculature and extracellular matrix (see. e.g., [43]), mimicking physiologic barriers for drug delivery existing in solid tumors. Importantly, lymphoma models demonstrate the accumulation of non-targeted nanocarriers (see e.g., [44]), which suggests the presence of the enhanced permeability and retention (EPR) effect, an unspecific tumor uptake of non-targeted nanoparticles and proteins with a molecular mass higher than 40 kDa [45]. Thus, lymphoma models could be used to assess the unspecific accumulation of macromolecules in vivo. The tumor uptake of both ^111^In-labeled conjugates was significantly (*p* < 0.05) higher in EpCAM-positive SKOV-3 than in EpCAM-negative Ramos xenografts 48 h after injection. The small uptake of ABD-fused DARPins in the Ramos xenografts is most likely due to the EPR-effect. Apparently, the contribution of the EPR-mediated uptake is small in the total uptake in SKOV-3 xenografts. Taken together with the in vitro data concerning the specificity (Figure 2) and high affinity (Table 2) of the interaction of [^111^In]In-Ec1-ABD and [^111^In]In-ABD-Ec1 with EpCAM-expressing cells, these data suggest that the tumor uptake of the constructs is EpCAM-specific.

**Figure 7:**
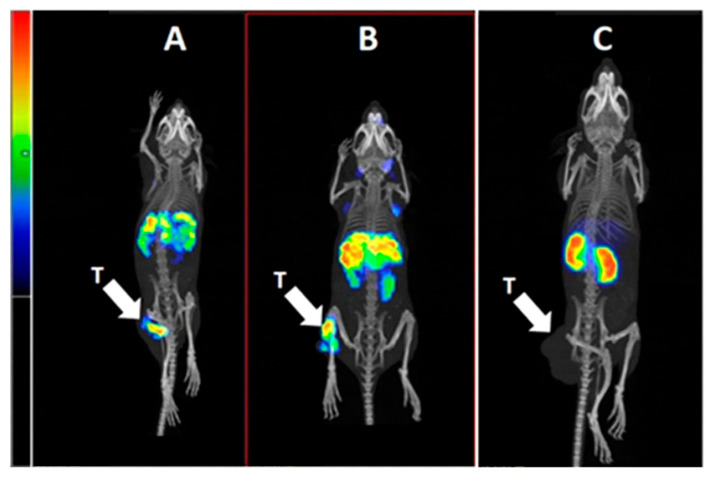
Imaging of distribution of (**A**) [^111^In]In-Ec1-ABD, (**B**) [^111^In]In-ABD-Ec1, and (**C**) [^111^In]In-Ec1 in mice bearing EpCAM-expressing SKOV-3 xenografts at 48 h after injection. An amount of 10 µg of labeled compound (1.5 MBq, 100 µL in 1% BSA in PBS) was injected into the tail vein. The arrow points to the tumor (T).

The results of the SPECT/CT imaging (Figure 7) of [^111^In]In-Ec1-ABD, [^111^In]In-ABD-Ec1, and [^111^In]In-Ec1 in BALB/c nu/nu mice bearing EpCAM-expressing SKOV-3 xenografts 48 h after injection confirmed the biodistribution data. A higher accumulation in tumors for [^111^In]In-ABD-Ec1 compared to other variants was observed. Renal uptake was dramatically lower for ABD-fused Ec1 variants (Figure 7A, B) than for non-ABD-fused Ec1 (Figure 7C).

Data for the non-ABD-fused variant are in agreement with the quick blood clearance obseved during the development of Ec1-based EpCAM imaging agents [23,24].

Overall, the fusion with ABD resulted in proteins with preserved specificity of binding to EpCAM (Figure 2). The binding strength decreased for ABD-fused variants, possibly due to steric hindrance. In the absence of albumin, this effect was similar for both variants; however, the affinity remained in the subnanomolar range. Adding albumin resulted in further affinity degradation, which was more pronounced in the case of N-terminal positioning (Table 2). This is the striking difference with HER2-binding G3 DARPins, where positioning of ABD at the C-terminus resulted in higher affinity in the presence of albumin [31]. The difference is not very surprising, as the binding geometry of G3 and Ec1 to their respective targets might be different. In addition, modeling revealed that there might be an interaction between G3 and ABD domains, which should be dependent on their positions in the fusion protein [46]. Apparently, this was not the case for Ec1-ABD.

Despite the reduced affinity, both ABD-fused variants enabled higher tumor uptakes than the non-fused variant (Figure 5 and Figure 7). It has to be noted that the extravasation rate is higher for smaller targeting agents [47]. However, the extravasation depends also on the concentration gradient between the blood and the tumor. In the case of rapidly excreted non-modified DARPin, a high clearance rate results in a rapid decrease in the gradient, as well as in reduced bioavailability. The reduced clearance rate of ABD-fused variants increases the probability of their extravasation in tumors and binding to the EpCAM expressed on malignant cells. Furthermore, the order of domains in ABD-fused constructs had a less pronounced effect on the blood concentration than on the tumor uptake. This suggests that the higher tumor uptake of [^111^In]In-ABD-Ec1 is determined by its higher affinity EpCAM.

Apparently, the size increase should be sufficient to prevent (or dramatically decrease) the glomerular filtration rate. Earlier, we evaluated a fusion of Ec1 with a deimmunized truncated variant of Pseudomonas exotoxin A (LoPE) [40]. This targeting protein has a molecular weight of 43 kDa, which should result in renal excretion. The blood concentration of this protein was below 0.4%ID/g four hours after injection. At the same time, the size increase resulted in diminished tumor uptake. The uptake in SKOV-3 xenografts was only 0.5% ID/g four hours after injection and decreased to below 0.1%ID/g at 48 h. The data concerning the rapid excretion of Ec1-LoPE are in agreement with the data for another fusion of anti-EpCAM DARPin (Ec4) with a truncated version of Pseudomonas exotoxin A (ETA’’) reported by Martin-Killias and co-authors [48]. The molecular weight of Ec4-ERA’’ was somewhat bigger, 59 kDa, but still below the cut-off for glomerular filtration. The plasma half-life of Ec4-ERA’’ was only 11.2 min.

Several alternative approaches to enhancing the blood residence time of DARPins have been evaluated by other researchers [9,29,49,50]. A direct coupling of albumin to the N-terminus of designed ankyrin repeat protein–cytotoxin fusion using Cu(I)-free click chemistry increased the blood half-life to 17 h, i.e., more efficiently than in this study [49]. However, this reduced the affinity for EpCAM three-fold; unfortunately, the effect on tumor uptake in vivo was not evaluated in that study. The effect of a bioorthogonal conjugation with a 20 kDa PEG polymer to the DARPin-PE40 toxin fusion was modest, and the elimination half-life of this variant was only 84 min [50]. Still, the PEG-coupled variant had stronger anti-tumor effect on HT-29 xenografts than the non-PEGylated version. These approaches require additional chemical conjugation steps, which increase the manufacturing costs. The gene engineering of bioavailability-modulating moieties might be more attractive from the clinical translation point of view [9,29]. The fusion of the long unstructured polypeptides PAS and XTEN appreciably extended the residence of Ec1 in the blood [29]. The use of a radioactive label, ^99m^Tc, showed that the effect of this fusion on the residence in blood was stronger than the effect of ABD fusion (half-lives 17.0 h and 20.6 h for the best variants, PAS900-Ec1 and XTEN864-Ec1, respectively). Still, the uptake of these proteins into tumors (10–11%ID/g) was similar to the uptake values from this study. It cannot be excluded that this is an effect of the bigger hydrodynamic radii of PASylated and XTENylated DARPins, which leads to less efficient extravasation and tumor uptake compared to ABD fusion. Thus, the use of a fusion with ABD is a promising approach to the modification of the pharmacokinetics of scaffold proteins. However, the effect of the fusion depends on the order of domains, which has to be taken into account during the development of such constructs. For example, a fusion of ABD at C-terminus of CEACAM5-targeting nanobody resulted in a modest increase of its blood concentration (2.3-fold) and its tumor uptake (1.6-fold) [51]. The magnitude of the blood half-life extension for the scaffold protein monobody (3.14 h) [52] after fusion of ABD at N-terminus was smaller than the extension for ABD-Ec1 in this study. It might be that the effect of the fusion with ABD would be stronger in these studies in the case of its positioning at the other terminus.

The development of targeted cytotoxic therapeutics is a long and complicated process. Initially, it was hoped to combine the cancer specificity of monoclonal antibodies and the cytotoxicity of bacterial toxins to enhance the efficacy of the cancer treatment [53]. The first variants of targeted toxins were prepared by chemical conjugation with immunoglobulins, but later variants where the cell-binding domain of a toxin was replaced by the Fv domain of an antibody were developed. The first approach to using DARPins was similar, and these scaffold proteins were fused with bacterial toxins [48,54]. However, the elevated immunogenicity of bacterial toxins resulted in the generation of neutralizing anti-drug antibodies in patients, which prevented multiple injections [55]. This was also the case for EpCAM-targeted immunotoxin MOC31PE, which caused neutralizing antibody development in all patients [56]. It turned out that immunotoxins might be efficient only in patients with hematologic malignancies with suppressed immune systems [53]. To enable the treatment of solid tumors, efforts towards the deimmunization of toxins, e.g., by identification and removal of B- and T-cell epitopes, are ongoing [55,57]. Still, the progress is limited, and even fusions of DARPins with deimmunized toxins elicit the formation of anti-drug antibodies, which limits their efficacy [54]. The immunogenicity problem is less challenging when using small-molecule drugs as cytotoxic agents. Currently, 15 antibody–drug conjugates have been approved for clinical use [58]. Thus, we put our efforts into the development of DARPin-based targeting systems capable of the delivery of small-molecule drugs. Importantly, the fusions of scaffold proteins with ABD are not immunogenic. Clinical studies have demonstrated that the fusion of an anti-IL12 affibody with ABD did not result in the formation of neutralizing antibodies after bi-weekly injections of high doses for several months [59].

DARPin and ABD scaffolds do not contain cysteine, and the introduction of a single cysteine by gene engineering creates a unique thiol group in the molecule that can be used for a well-controlled site-specific coupling of a pendant group by sulfhydryl-directed conjugation chemistry. In this study, we utilized this for the coupling of the DOTA chelator for radiolabeling. However, we demonstrated that this coupling strategy might be used for the attachment of a variety of drugs, such as auristatin and maytansine maleimide derivatives [60,61,62] to scaffold protein–ABD fusions. Both in vitro and in vivo studies have demonstrated that these constructs possess an anti-tumor effect dependent on a target-specific delivery of a drug. The effect of such drug conjugates was much stronger than the effect of fusions without attached drugs or homologous control drug conjugates unable to bind molecular targets.

Apparently, the fusion or conjugation with ABD might be applied not only to DARPins or other scaffold proteins. For example, peptide–drug conjugates (PDCs) have become the emerging targeted anti-cancer therapeutics [63,64]. However, the rapid renal clearance of peptides causes their short residence in circulation and suboptimal targeting, meaning they require frequent administration [63]. We have shown that the conjugation of peptide RM26 with ABD enables an extension of its residence in blood and better delivery to tumor models [65]. However, that study also demonstrated the need for careful optimization of the construct’s architecture.

## 3. Conclusions

Fusion with the ABD increased the blood residence time of the anti-EpCAM DARPin, resulting in higher uptake in tumors due to the higher bioavailability of the targeting agent compared with the non-ABD-fused counterpart. The fusion reduces the glomerular filtration of these DARPins, resulting in a dramatic reduction of renal reabsorption and retention. The effect of fusion with ABD on the biodistribution and targeting properties of the DARPin Ec1 depends on the order of the domains in the construct. The positioning of ADB at the N-terminus provided double the tumor uptake and created a better precondition for the targeted delivery of cytotoxic payloads.

## 4. Materials and Methods

### 4.1. General

The chemicals were purchased from Sigma-Aldrich, Sweden AB (Stockholm, Sweden). Buffers were prepared using high-quality Milli-Q water and purified from metal contaminations using Chelex 100 resin (Bio-Rad Laboratories, Hercules, CA, USA). [^111^In]InCl_3_ was purchased from Curium Pharma (Stockholm, Sweden). The NAP-5 size-exclusion column used for purification was from GE Healthcare.

Radioactivity was measured using an automated gamma-spectrometer with an NaI (TI) detector (2480 Wizard, Wallac, Finland). A Cyclone Storage Phosphor System (CR-35 BIO Plus, Elysia-Raytest, Bietigheim-Bissingen, Germany) and OptiQuant image analysis software v.2.0 (PerkinElmer, Waltham, MA, USA) were used for measuring the radioactivity distribution on instant thin-layer chromatography (iTLC) strips.

The ovarian cancer cell lines SKOV-3 and OVCAR-3, both from the American Type Culture Collection (ATCC, Manassas, MA, USA), were used. The Ramos lymphoma cell line (ATCC) was used to establish EpCAM-negative xenografts. Cells were cultured in Roswell Park Memorial Institute (RPMI) 1640 medium (Sigma-Aldrich, St. Louis, MO, USA), supplemented with fetal bovine serum (10%), Lglutamine (2 mM), penicillin (100 IU/mL), and streptomycin (100 mg/mL). Human serum albumin (HSA) was purchased from Sigma-Aldrich, Sweden AB (Stockholm, Sweden).

### 4.2. Statistical Analysis

To determine significant differences (*p* < 0.05), data on in vitro studies were analyzed by unpaired 2-tailed *t*-test using GraphPad Prism (version 10.1.0 for Windows; GraphPad Software LLC, San Diego, CA, USA). Biodistribution data were analyzed by ANOVA using GraphPad Prism (version 10.4.1 for Windows; GraphPad Software LLC, San Diego, CA, USA) to determine significant differences (*p* < 0.05).

### 4.3. Production, Purification, and Characterization of ABD-Fused DARPin Ec1 Variants and Ec1 and Conjugation of DOTA Chelator

The ABD-fused DARPins (Supplementary Figure S1) were produced according to the protocol described earlier [31]. The nucleotide sequences of ABD_035_ and DARPin Ec1 were deduced from [10,66]. For conjugation of ABD-fused Ec1 variants to 1,4,7,10-tetraazacyclododecane-1,4,7-Tris-acetic acid-10-maleimidoethylacetamide (mal-DOTA) (MW = 526.4 mg/mmol), (HE)_3_-Ec1-ABD-E_3_C, or (HE)_3_-ABD-Ec1-E_3_C (1 mg, 41 nmol, 220 μL in PBS, pH 8) were incubated with a 200-fold molar excess of dithiothreitol (DTT) (1.28 mg, 8.2 μmol, 26 μL in PBS, pH 8.0, final DTT concentration 34 mM) for 30 min at 40 °C. To remove DTT, the reaction mixture was purified using a NAP-5 column, pre-equilibrated with degassed 0.2 M ammonium acetate (NH_4_OAc), pH 6.5. Next, the fractions containing the protein (0.9 mg, 37 nmol, 470 μL) were incubated with a 5-fold molar excess of maleimide-DOTA (146 μg, 185 nmol, 29 μL of 5 mg/mL in 0.2 M NH_4_OAc, pH 6.5) at 40 °C for 1 h. To remove the unconjugated chelator, the reaction mixture was purified using a NAP-5 column with 0.2 M NH_4_OAc, pH 5.5. The protein concentration was measured using a DS-11 spectrophotometer (DeNovix, Wilmington, DE, USA). The (HE)_3_-Ec1-ABD-E_3_C and (HE)_3_-ABD-Ec1-E_3_C variants conjugated to DOTA (termed Ec1-ABD and ABD-Ec1, respectively) were stored in 0.2 M NH_4_OAc (pH 5.5) at −20 °C before labeling with radiometals. The molecular masses of the proteins were determined by high-resolution mass spectrometry using a 6520 Accurate-Mass Q-TOF LC/MS (Agilent Technologies, Santa Clara, CA, USA). The purity of the constructs was confirmed using LC-MS. The control, non-ABD-fused DARPin Ec1 containing C-terminal cysteine was produced and conjugated with DOTA as described by Deyev and co-workers [24].

### 4.4. Radiolabeling of DARPin Ec1 with ^111^In and In Vitro Stability

Labeling of DARPin Ec1 variants with indium-111 was performed at 60 °C as described previously [24]. The in vitro stability test of Ec1 variants labeled with ^111^In was performed by incubating a fresh fraction of purified radioconjugates with a 1000-fold molar excess of EDTA. The three control samples were incubated in PBS. Three aliquots per sample were analyzed.

Radio-iTLC data were confirmed using reverse-phase HPLC analysis. This analysis was performed using an Elite LaChrom system (Hitachi, VWR, Darmstadt, Germany) equipped with a radiation flow detector (Bioscan, Washington, DC, USA) and an analytical column (Vydac RP C18 column, 300 Å; 3 × 150 mm; 5 µm). The HPLC conditions were as follows: A = 10 mM TFA/H_2_O, B = 10 mM TFA/acetonitrile, UV-detection at 220 nm, gradient elution: 0–15 min at 5% to 70% B, 15–18 min at 70% to 95% B, 19–20 min at 5% B, and a flow rate was 1.0 mL/min.

### 4.5. In Vitro Studies

The binding specificity to EpCAM in SKOV-3 and OVKAR3 cells was evaluated using a saturation test described previously for non-ABD-fused Ec1 [26]. Briefly, a large molar excess of unlabeled Ec1 (200 nM in 500 µL) was added to control cells to saturate EpCAM, followed by the addition of radiolabeled DARPin radioconjugate (4 nM in 500 µL) to both groups (2 nM final concentration).

The binding kinetics of the radioconjugates to living cells was measured using a LigandTracer Yellow instrument (Ridgeview Instruments AB, Uppsala, Sweden), as described earlier [67]. The association rate was measured at 9 and 27 nM concentrations of the radiolabeled conjugate. Additionally, the experiment was performed in the presence of 100 nM HSA in complete media to estimate the impact of albumin on the binding to the molecular target. The measurements were performed in duplicate. The data were analyzed using InteractionMap software (Ridgeview Instruments AB, Uppsala, Sweden, https://www.ligandtracer.com/product/interaction-map/#tab-description, accessed 28 May 2025) to calculate the association rate, the dissociation rate, and the dissociation constant at equilibrium (K_D_).

The cellular processing of Ec1 radioconjugates by ovarian cancer cell lines during continuous incubation was studied using an acid-wash method, as described for non-ABD-fused constructs [25]. Three dishes per time point were used. Radioconjugates (2 nM) were added to the dishes and cells were incubated at 37 °C in a humidified incubator for 1, 2, 4, 8, and 24 h. To investigate the influence of HSA on cellular processing, the experiment was performed in the presence of HSA (100 nM).

### 4.6. In Vivo Studies

The blood kinetics of ABD-fused Ec1 variants were studied in female NMRI mice. The average animal weight was 35 ± 2 g at the time of the experiment. The animals (a group of four mice per data point) were euthanized at 4, 24, and 72 h after the injection of 30 kBq/0.3 nmol (in 100 µL of 1% BSA in PBS per mouse) of [^111^In]In-Ec1-ABD and [^111^In]In-ABD-Ec1. At each time point, blood was collected and weighed. The blood activity concentration was measured, and decay correction of activity was performed. The half-life of activity in blood was calculated using GraphPad Prism (nonlinear regression, one phase decay, version 10.4.1 for Windows; GraphPad Software LLC, San Diego, CA, USA).

Female BALB/c nu/nu mice were supplied by Scanbur A/S (Karlslunde, Denmark) and had an adaptation period of one week before the start of experimental procedures. For the implantation of EpCAM-positive xenografts, 10^7^ SKOV-3 cells were subcutaneously injected into the hind legs of mice. To establish EpCAM-negative xenografts, 5 × 10^6^ Ramos lymphoma cells were implanted. The animal biodistribution measurements were performed three weeks after implantation. A group of 4 mice was used for each time point. The average animal weight was 19.3 ± 1.5 g at the time of the experiment.

To compare the biodistribution of ABD-fused Ec1 variants labeled with ^111^In, two groups of mice with EpCAM-positive SKOV-3 xenografts were intravenously injected (20 kBq, 0.3 nmol, 100 µL of 1% BSA in PBS per mouse). The average tumor weight was 0.71 ± 0.35 g. The mice were euthanized 48 after injection by overdosing anesthesia followed by heart puncture. The tumors and organs were collected, weighed, and their activity was measured. The uptake values were calculated as the percentage of the injected dose per gram of the sample (% ID/g). To evaluate the effect of the fusion of DARPin Ec1 with ABD on biodistribution, one group of mice with SKOV-3 xenografts was injected with non-ABD-fused [^111^In]In-Ec1 (20 kBq, 0.3 nmol, in 100 µL of 1% BSA in PBS per mouse). The average tumor weight was 1.01 ± 0.59 g. The measurement was performed 48 h after injection.

Two groups of mice with EpCAM-negative Ramos xenografts were injected with [^111^In]In-Ec1-ABD and [^111^In]In-ABD-Ec1 (20 kBq, 0.3 nmol, in 100 µL of 1% BSA in PBS) to test if the tumor uptake depended on the level of EpCAM expression. The average Ramos tumor weight was 0.56 ± 0.27 g. The measurement was performed 48 h after injection.

Whole-body SPECT/CT imaging was performed to obtain a visual confirmation of the biodistribution data. Mice with SKOV-3 xenografts were injected with equal activities (1.4 MBq) of the radioconjugates [^111^In]In-Ec1-ABD, [^111^In]In-ABD-Ec1, and [^111^In]In-Ec1. The mice were imaged at 48 h after injection using a nanoPECT/CT scanner (Mediso Medical Imaging Systems, Budapest, Hungary) as described earlier [24].

## Figures and Tables

**Table 1:** Radiolabeling results and in vitro stability.

	Radiochemical Yield, %	Radiochemical Yield After EDTA Treatment, %	Isolated Yield, %	Radiochemical Purity, %	% Stability in PBS (1 h at 37 °C)	% Stability (×1000 EDTA, 1 h at 37 °C)
Ec1-ABD	92 ± 7	79 ± 18	46 ± 16	99 ± 2	90.7 ± 0.6	91.7 ± 1.5
ABD-Ec1	61 ± 24	52 ± 23	38 ± 18	100 ± 1	96.9 ± 2.6	94.8 ± 1.8
Ec1	60	52	20	96	-	-

**Table 2:** Results of InteractionMap analysis of [^111^In]In-Ec1-ABD and [^111^In]In-ABD-Ec1 binding to EpCAM-expressing SKOV-3 ovarian cancer cells in the presence and absence of HSA (100 nM). * Data are taken from ref. [24].

Compound	k_a_ (1/(M*s)) (10^3^)	k_d_ (1/s) (10^−6^)	K_D_ (nM)
[^111^In]In-Ec1-ABD (−HSA)	7.4 ± 2.7	3.5 ± 0.6	0.523 ± 0.212
[^111^In]In-Ec1-ABD (+HSA)	10.2 ± 5.8	21.2 ± 4.0	2.6 ± 1.9
[^111^In]In-ABD-Ec1(−HSA)	8.0 ± 3.4	2.8 ± 0.5	0.368 ± 0.086
[^111^In]In-ABD-Ec1(+HSA)	8.0 ± 6.6	7.3 ± 6.5	0.863 ± 0.103
[^111^In]In-Ec1 *	-	-	0.21 ± 0.01

## Data Availability

The data presented in this study are available upon request from the corresponding author.

## Supplementary Materials

The following supporting information can be downloaded at: https://www.mdpi.com/article/10.3390/ijms26115236/s1.

## Abbreviations

kDa, Kilodalton; keV, Kilo-electronvolt; IMAC, Immobilized metal affinity chromatography; LC-MS, Liquid chromatography–mass spectrometry; HPLC, High-performance liquid chromatography; SPECT/CT, Single-photon emission computed tomography/computed tomography.
