# Supplementary material for: Targeting of Epithelial Cell Adhesion Molecule-Expressing Malignant Tumors Using an Albumin-Binding Domain-Fused Designed Ankyrin Repeat Protein: Effect of the Molecular Architecture
Source: Int J Mol Sci. 2025 May 29;26(11):5236. doi: 10.3390/ijms26115236 (PMC12154114; doi:10.3390/ijms26115236)
Supplement: Supplementary file 1 [file ijms-26-05236-s001.zip › ijms-3586413-supplementary.pdf]

Supplementary Figure S1.

HEHEHE-DARPin Ec1-Linker-ABD035-EEEC: Calc. Mw 24661,55 Da

SHEHEHEGSDLGKKLLEAARAGQDDEVRILVANGADVNAYFGTTPLHLAAAHGRLEIVEVLLKNGADVNAQDVWGITPLHLAAYNGHLEIVEVLLKYGADVNAHDTRGWTPLHLAAINGHLEIVEVLLKNVADVNAQDRSGKTPFDLAIDNGNEDIAEVLQKAAKLN**GSSSGSSSGSSS**LAEAKVLANRELDKYGVSDFYKRLINKAKTVEGVEALKLHILAALPGSEEEC

HEHEHE-ABD035-Linker-DARPin Ec1-EEEC: Calc. Mw 24661,55 Da

SHEHEHEGSLAEAKVLANRELDKYGVSDFYKRLINKAKTVEGVEALKLHILAALP**GSSSGSSSGSSS**DLGKKLLEAARAGQDDEVRILVANGADVNAYFGTTPLHLAAAHGRLEIVEVLLKNGADVNAQDVWGITPLHLAAYNGHLEIVEVLLKYGADVNAHDTRGWTPLHLAAINGHLEIVEVLLKNVADVNAQDRSGKTPFDLAIDNGNEDIAEVLQKAAKLNGSEEEC

HEHEHE-DARPin Ec1-EEEC: Calc. Mw 18614,71 Da

SHEHEHEGSDLGKKLLEAARAGQDDEVRILVANGADVNAYFGTTPLHLAAAHGRLEIVEVLLKNGADVNAQDVWGITPLHLAAYNGHLEIVEVLLKYGADVNAHDTRGWTPLHLAAINGHLEIVEVLLKNVADVNAQDRSGKTPFDLAIDNGNEDIAEVLQKAAKLNGSEEEC

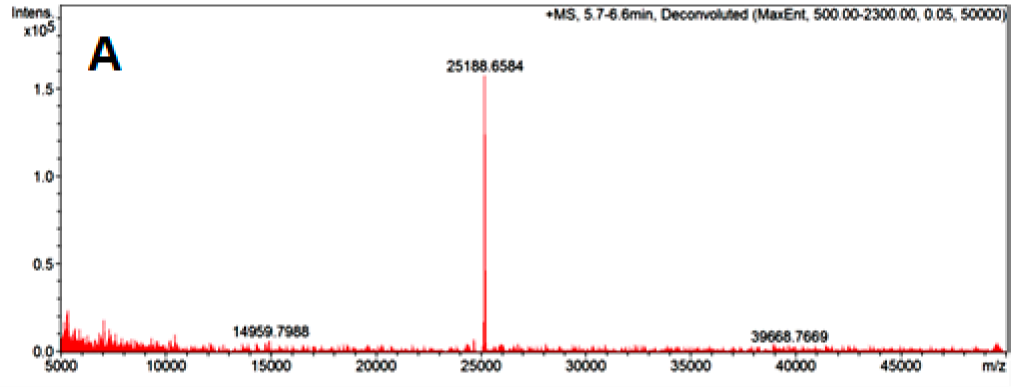

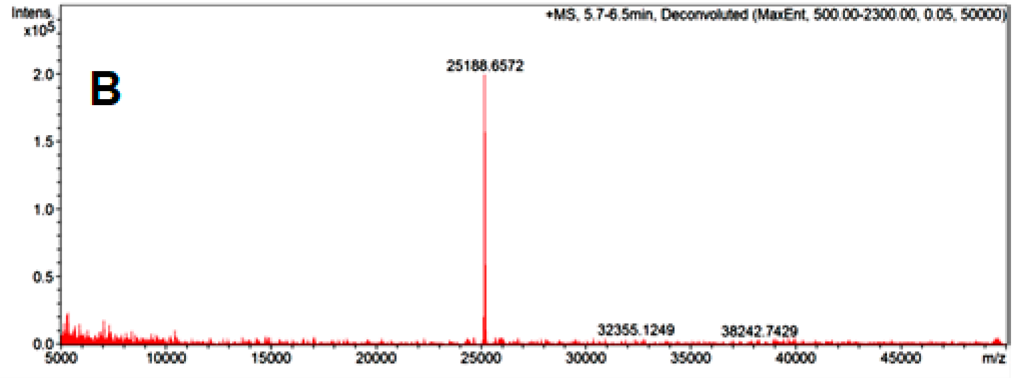

Supplementary Figure S2. Mass spectra of DOTA-conjugated variants: ABD-Ec1-DOTA (A) and Ec1-ABD-DOTA (B) Calculated molecular weight was 25189,6 kDa, found weight 25188.7 kDa for both conjugates.

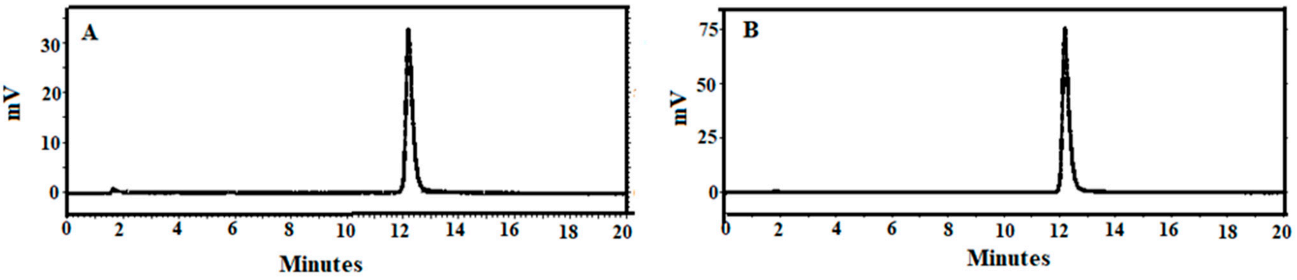

Supplementary Figure S3. Reversed-phase radio-HPLC chromatograms of (A) [^{111}In]In-Ec1-ABD and (B) [^{111}In]In-ABD-Ec1. The retention times (Rt) are expressed in minutes.

Supplementary Table S1. Comparative biodistribution of DARPin Ec1 variants [^{111}In]In-Ec1-ABD, [^{111}In]In-ABD-Ec1 and non-ABD-fused [^{111}In]In-Ec1 as a control conjugate in BALB/C nu/nu mice bearing EpCAM-expressing SKOV-3 xenografts at 48 h after injection. Uptake is expressed as %ID/g and presented as average value from 4 mice ±SD.

**Site**	**[^{111}In]In-Ec1-ABD**	**[^{111}In]In-ABD-Ec1**	**[^{111}In]In-Ec1**
Blood	2.27±0.23[a,c]	3.76±0.81[b,c]	0.03±0.01[a,b]
Lung	1.82±0.38[a,c]	2.99±0.51[b,c]	0.23±0.05[a,b]
Liver	11.77±3.42[a]	12.45±2.90[b]	3.88±0.59[a,b]
Spleen	4.16±0.92[a]	4.47±0.69[b]	1.56±0.29[a,b]
Pancreas	0.62±0.07[a,c]	0.92±0.20[b,c]	0.29±0.08[a,b]
Small intestine	1.00±0.24[a]	1.18±0.32[b]	0.23±0.05[a,b]
Stomach	0.56±0.08[c]	0.95±0.29[b,c]	0.30±0.05[b]
Kidney	9.58±1.16[a]	12.09±1.05[b]	168.02±28.03[a,b]
Tumor	6.56±1.22[a,c]	13.19±1.48[b,c]	2.72±0.45[a,b]
Muscle	0.58±0.19[a,c]	1.00±0.32[b,c]	0.13±0.05[a,b]
Bone	0.87±0.11[a,c]	1.54±0.15[b,c]	0.53±0.03[a,b]
GI tract*	1.21±0.13	2.04±0.15	0.57±0.34
Carcass*	19.18±3.69	27.04±2.38	4.86±0.92

[a] Significant ($p<0.05$) difference between [^{111}In]In-Ec1-ABD and [^{111}In]In-Ec1;

[b] Significant ($p<0.05$) difference between [^{111}In]In-ABD-Ec1 and [^{111}In]In-Ec1;

[c] Significant ($p<0.05$) difference between [^{111}In]In-Ec1-ABD and [^{111}In]In-ABD-Ec1.

* Data for gastrointestinal (GI) tract with content and carcass are presented as % of injected dose per whole sample. ANOVA test (Bonferroni's multiple comparisons test) was performed to test significant ($p<0.05$) difference.
